# A Deeper Insight in Metal Binding to the hCtr1 N-terminus Fragment: Affinity, Speciation and Binding Mode of Binuclear Cu^2+^ and Mononuclear Ag^+^ Complex Species

**DOI:** 10.3390/ijms23062929

**Published:** 2022-03-08

**Authors:** Antonio Magrì, Giovanni Tabbì, Irina Naletova, Francesco Attanasio, Giuseppe Arena, Enrico Rizzarelli

**Affiliations:** 1 Institute of Crystallography, National Council of Research, CNR, S.S. Catania, Via P. Gaifami 18, 95126 Catania, Italy; antonio.magri@ic.cnr.it (A.M.); giovanni.tabbi@ic.cnr.it (G.T.); irina.naletova@ic.cnr.it (I.N.); 2 Consorzio Interuniversitario per la Ricerca dei Metalli nei Sistemi Biologici, Via Ulpiani 27, 70126 Bari, Italy; 3 Department of Chemical Sciences, University of Catania, Viale A. Doria 6, 95125 Catania, Italy; garena@unict.it

**Keywords:** Ctr1, model transporter, copper, silver, speciation, affinity, reductase, antibody recognition

## Abstract

Ctr1 regulates copper uptake and its intracellular distribution. The first 14 amino acid sequence of the Ctr1 ectodomain Ctr1_(1-14)_ encompasses the characteristic Amino Terminal Cu^2+^ and Ni^2+^ binding motif (ATCUN) as well as the bis-His binding motif (His5 and His6). We report a combined thermodynamic and spectroscopic (UV-vis, CD, EPR) study dealing with the formation of Cu^2+^ homobinuclear complexes with Ctr1_(1-14)_, the percentage of which is not negligible even in the presence of a small Cu^2+^ excess and clearly prevails at a M/L ratio of 1.9. Ascorbate fails to reduce Cu^2+^ when bound to the ATCUN motif, while it reduces Cu^2+^ when bound to the His5-His6 motif involved in the formation of binuclear species. The histidine diade characterizes the second binding site and is thought to be responsible for ascorbate oxidation. Binding constants and speciation of Ag^+^ complexes with Ctr1_(1-14)_, which are assumed to mimic Cu^+^ interaction with N-terminus of Ctr1_(1-14)_, were also determined. A preliminary immunoblot assay evidences that the anti-Ctr1 extracellular antibody recognizes Ctr1_(1-14)_ in a different way from the longer Ctr1_(1-25)_ that encompasses a second His and Met rich domain.

## 1. Introduction

Copper is an essential element for life that is needed for all cellular systems of both prokaryotic and eukaryotic organisms [1] Cu^+^/Cu^2+^ redox process can be detrimental to cells, as it may result in the production of different reactive oxygen, nitrogen and carbon species that are thought to be the cause of different disorders [2,3].

A defective absorption of the metal ion is linked to a series of pathologies, while an excessive copper accumulation may cause degenerative disorders [4,5].

Thus, it is not a surprise that copper homeostasis is strictly controlled in all eukaryotic organisms. To this end, a protein network is specifically devoted to the regulation of intracellular Cu^+^ levels. The membrane copper transporter 1 (Ctr1), which belongs to the SLC31 family of solute carriers [6] and possesses high specificity and affinity for Cu^+^, is the most important protein transporting copper into the cell [7] Ctr1 in concert with Cu^+^-transporting ATPase pumps (ATP7A and ATP7B) [8], which are localized in the intracellular membrane and harmonize copper trafficking by regulating copper uptake, intracellular distribution and eventually its export from the cell [9]. Human Ctr1 (hCtr1) consists of 190 amino acids and shares a large homology with yeast Ctr1 (yCtr1), though it is characterized by peculiar differences. Both hCtr1 and yCtr1 have in common three transmembrane domains (TMDs), the second of which contains a conserved MXXXM motif, which is deemed critical for copper uptake [10]. Ctr1 extracellular domain is composed of sixty-six aminoacids containing two methionine motifs (Mets) and a mostly unstructured N-terminus that is thought to be responsible for copper binding. However, differently from the yCtr1, the hCtr1 domain is rich in histidine and methionine residues [11]. Recently, both crystallographic [12,13] and biochemical findings [14] have provided some evidence indicating that Ctr1 forms a homotrimer with a peculiar structure that closely recalls an ion channel. The nine TMDs of the trimer are arranged in such a way as to form a cone-shaped structure that is well-suited to a passive transport of the metal ion. hCtr1 shows a high selectivity for Cu^+^ over Cu^2+^ [15]; but it is not clear yet whether in mammals the copper reduction process takes place before (implying STEAP4) [16] or once the metal ion is transferred from its complex with human serum albumin (HSA) to hCtr1 [17]. It has been demonstrated that, in yeast, (i) yCtr1 necessitates the presence of metallo-reductases (Fre family) on cell surfaces [18,19], and (ii) the addition of a reducing agent (ascorbate) to cell cultures increases copper acquisition [20,21,22]. Recently, it has been suggested that hCtr1 might act as a copper reductase by interacting with extracellular ascorbate that is present at a millimolar concentration level; specifically, there is evidence showing that the first fourteen aminoacids of hCtr1 N-terminal domain (Ctr1_(1-14)_) (H_2_N-MDHSHHMGMSYMDS-NH_2_) are crucial for the binding as well as the reduction of copper [23].

Besides the characteristic Amino Terminal Cu^2+^ and Ni^2+^ binding motif (ATCUN) [24], which involves a 4N (1 amino nitrogen, 1 imidazole nitrogen, 2 amide deprotonated nitrogen atoms) Cu^2+^ coordination arrangement, hCtr1_(1-14)_ possesses a bis-His binding motif (His5 and His6), one amino acid distant from ATCUN.

The contemporary presence of both the ATCUN sequence (MDH) and the bis-His motif confers to the hCtr1 mimicking fragment a high affinity not only for Cu^2+^ but also for Cu^+^; notably, Cu^+^ affinity for yCtr1, which contains only Mets-motifs specific for Cu^+^, is six orders of magnitude smaller [25,26]. The bis-His motif assists the reduction of Cu^2+^ [27] and should stabilize Cu^+^ complex species [28]. Cu^2+^ and Cu^+^ binding mode and affinity to hCtr1_(1-14)_ have been widely investigated by different research groups with contrasting results in some cases [29]. Based on X-ray absorption spectroscopy (XAS) data, Haas et al. indicated that Cu^+^ binds to this model peptide with a 2N1O1S coordination arrangement that involves the imidazole nitrogen atoms of His5 and His6 of the bis-His sequence, a sulfur atom of a methionine residue and the carbonyl oxygen of the His5-His6 amide bond [27]. Shenberger et al. suggested that His3, His5 and His6 may all be involved in Cu^2+^ binding and concluded that Cu^2+^ binds to hCtr1 via a 3N1O arrangement [29] at odds with the 4N coordination mode proposed by Haas et al. for Cu^2+^ [23].

By combining UV-vis and calorimetric experiments, Du et al. investigated an N-terminal model containing fifty-five aminoacids [30]. They concluded that two Cu^2+^ ions bind to this fragment and that the ATCUN and the bis-His motifs may constitute two distinct Cu^2+^ binding sites with binding constants that differ by an order of magnitude (3.7 × 10^9^ M^−1^ and 2.6 × 10^8^ M^−1^). By contrast, Haas et al. reported only one constant for the binding of Cu^2+^ to a fourteen aminoacids model of the N-terminal fragment with a somewhat different affinity constant value (1 × 10^11^ M^−1^) [23]. Noteworthy is the fact that, though both groups provide converging evidence on Cu^2+^ binding to the MDH site, the stability constant values are much lower than those reported for Cu^2+^ complexes with the high affinity N-terminus of human serum albumin (HSA) [31]. Perhaps the underestimation of some of these values results from neglecting the competition of the buffer [32]. The issue concerning the metal ion transfer from HSA to Ctr1_(1-14)_ has been overcome by recent investigations [17,32] reporting more reliable affinity values for the binding of Cu^2+^ to Ctr1_(1-14)_, which might account for the metal ion transfer from HSA.

Different affinity constant values have been reported for the Cu^+^ complexes with the N-terminus of the Ctr1 ectodomain, which can perhaps ascribed to the different lengths of the investigated polypeptides. Specifically, incubating hCtr1_(1-46)_ with different amounts of Cu^2+^ or Cu^+^ results in the coordination of up to three or six Cu^2+^ or Cu^+^ ions, respectively [33]. The log K value determined for Cu^+^-hCtr1_(1-46)_ is of the same order of magnitude of that obtained for Cu^+^-hCtr1_(1-55)_ [30] (14.79 vs. 14.92), while it is some six orders of magnitude larger than that of Cu^+^-hCtr1_(1-14)_ [23]. Such a pronounced difference strongly suggests that the residues contained in the segment 15-46 contribute to the binding of the metal ion and account for the different stability constants found, which, in turn, may be ascribed to the involvement of His and Met motifs in the longer peptide segment (hCtr1_(1-46)_) [33]. Additionally, Cu K-edge X-ray absorption spectroscopy results support a structure containing a tetrahedrally arranged Cu^+^ with a 2N2S set of donor atoms. Tandem ESI-MS results indicate that the coordination sites are provided by the first His-rich and the second Met-rich motifs of the segment, though different Cu^+^-binding arrangements may still be possible for the full-length protein that forms a trimeric cone-shaped pore [13].

The ^1^H NMR shifts detected in the proximity of the His and Met residues indicate that the His and Met residues are heavily involved in the coordination to Cu^+^ [29], which corroborates Haas et al.’s previous reports [27,28] that support a 2N1O1S Cu^+^ coordination in Ctr1_(1-14)_.

Defining the arrangement around copper is of crucial importance, as it would impact on the ease of reduction of Cu^2+^ ions coordinated to the N-terminus ATCUN motif. It has been shown that Cu^2+^ reduction by ascorbate is faster when hCtr1 model peptides contain the bis-His motif [23] and is even significantly faster for the trimer containing three MDHSHH motifs than for the analogous monomer containing a single MDHSHH unit [34]. Interestingly, Cu^2+^ ions bound to MDHS motifs and lacking the bis-His units are also less susceptible to reduction. This is in keeping with recent reports [35,36] that indicate that Cu^2+^ bound to ATCUN via a 4N coordination mode is not reduced, though it is also suggested that a species where the metal ion involves the binding of an amine and an imidazole nitrogen is responsible for the reduction process [36]. Remarkably, by competition experiments with bicinchoninic acid, it has been shown that the trimer containing the MDHSHH units are much better competitors than those with the MDHS motifs, thus indicating that the bis-His sequence is mainly responsible for Cu^+^ binding.

However, despite the various attempts made in the last couple of decades to shed light on the structure of the coordination site(s), the way in which Cu^+^ crosses Ctr1 pore as well as Ctr1 selectivity towards Cu^+^ and Cu^2+^ still remain unclear. The research results that appear in the literature so far do not fully clarify the following issues: (i) the reason why ascorbate fails to reduce Cu^2+^ bound to the ATCUN motif in short peptides containing the MDH sequence only [35,36] whilst the same agent reduces the ATCUN bound Cu^2+^ in Ctr1_(1-14)_ having both the MDH sequence and a histidine diade (His5 and His6); (ii) the dynamics of the transfer of reduced copper from the ATCUN site to the His5-His6 motif and the stabilizing effect on the resulting Cu^+^ complex species by the histidine diade system; (iii) the formation of Cu^2+^ homobinuclear species in Ctr1_(1-14)_.

In an attempt to answer the questions above, we report a combined thermodynamic and spectroscopic (UV-vis, CD, EPR) study dealing with the formation of binuclear species of Cu^2+^ with Ctr1_(1-14)_ as well as the binding constants and speciation of Ag^+^ complexes with Ctr1_(1-14)_ that are assumed to mimic Cu^+^ interaction with the N-terminus of Ctr1_(1-14)_. We have carried out experiments to identify the species responsible for Cu^2+^ reduction by ascorbate. Lastly, the effect of the metal ion interaction on the conformation of the N-terminus ectodomain has also been investigated by an in vitro assay.

## 2. Results and Discussion

### 2.1. Ctr1_(1-14)_ Can Bind Two Cu^2+^ Equivalents

The affinity of Cu^2+^ complexes with Ctr1_(1-14)_ has been studied by different groups [17,37] with different experimental approaches that have a significant impact on the stability constant values [17]. Stefaniak et al. have explored a 1:1 Cu^2+^/Ctr1_(1-14)_ ratio and obtained the stability constant values and the related speciation supporting the ability of the N-terminus of hCtr1 to sequester Cu^2+^ from human serum albumin [17]. The presence of a His motif in the Ctr1_(1-14)_ sequence and clues coming from studies on Ctr1_(1-14)_ as well as homologous shorter and longer peptides [29,30,34] prompted us to investigate the system in detail to specifically see whether Cu^2+^ interaction with Ctr1_(1-14)_ also resulted in the formation of binuclear copper complex species.

**Figure 1:**
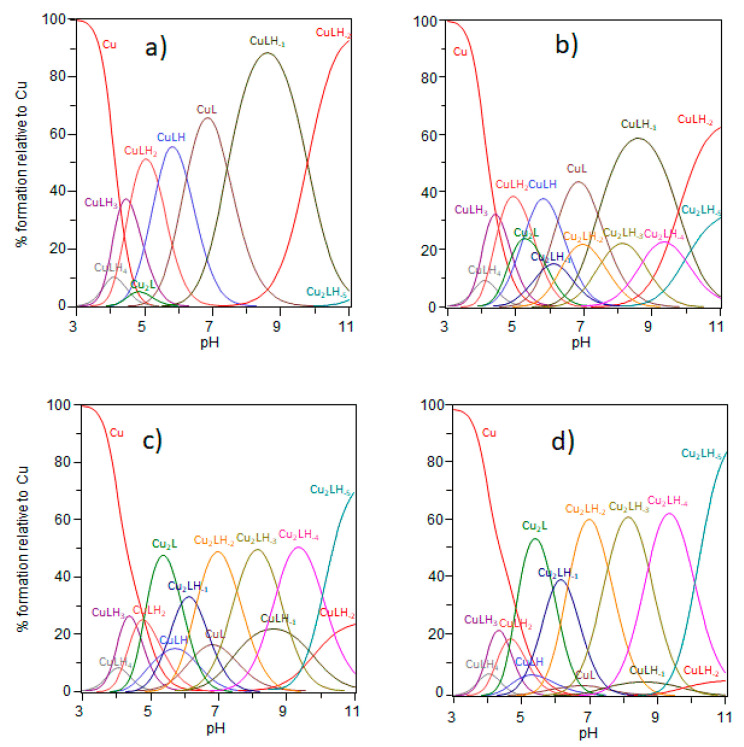
Species distribution for the Cu^2+^- Ctr1_(1-14)_ system. C_Ctr1_ = 5 × 10^−4^ mol dm^−3^; Cu^2+^/Ctr1_(1-14)_ concentration ratio is 0.9, 1.2, 1.6 and 1.9 for (**a**–**d**), respectively.

Ctr1_(1-14)_ has seven sites that can be protonated (i.e., two carboxylate side chains, three nitrogen atoms from imidazole rings, the terminal NH_2_ group and the phenolate group of Tyr side chain); accordingly, the fully protonated ligand is defined as [H_7_L]. The protonation constants values determined by us (Table S1) are in good agreement with those obtained in a previous work [17]. Cu^2+^ affinity for Ctr1_(1-14)_ was investigated potentiometrically by exploring a Cu^2+^/Ctr1_(1-14)_ concentration ratio ranging from 0.9 to 1.9; such a concentration range is wider than that investigated previously by others [17]. The ‘best’ set of stability constants is reported in Table 1 together with the values determined by Stefaniak et al. [17], while the species distribution resulting from our data at different Cu^2+^/Ctr1_(1-14)_ ratios is shown in Figure 1.

**Figure 2:**
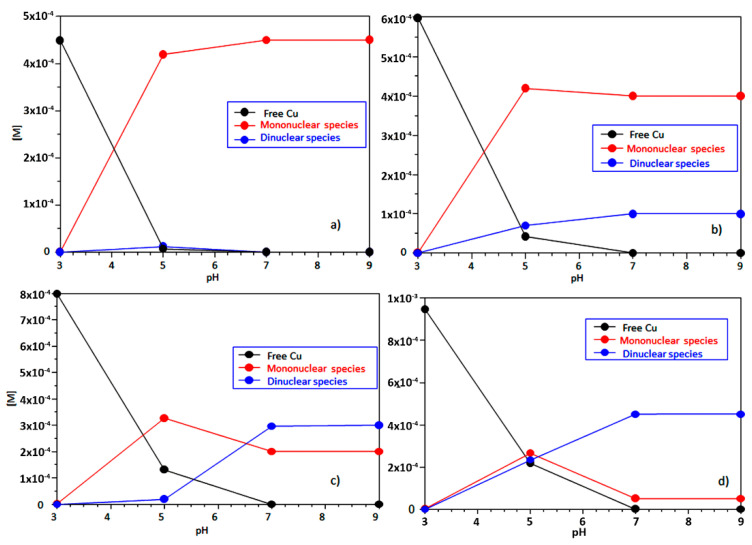
Total concentration of Cu^2+^-Ctr1_(1-14)_ mononuclear and binuclear complexes species at Cu^2+^/Ctr1_(1-14)_ concentration ratios equal to 0.9, 1.2, 1.6 and 1.9 for (**a**–**d**), respectively.

Since hCtr1 is also found in some intracellular areas (e.g., lysosomes) where pH is acidic, we started our study from pH values as low as 3 (Figure 1) [14,38]. The set of mononuclear species detected in the present study as well as the values of their stability constants mirror those reported by Stefaniak et al. [17], who restricted their investigation to the 0.4–0.9 Cu^2+^/Ctr1_(1-14)_ range, however; the coordination mode of mononuclear species has been discussed previously [17]. Noteworthy is the fact that, in addition to mononuclear species, we have also detected the formation of copper binuclear species, the percentage of which is not negligible even in quasi-equimolar concentrations of Cu^2+^ and Ctr1_(1-14)_ (Figure 1b). Likely, this arises from the wider concentration ratio explored in our study. In order to give a better perception of their significance, we have computed the total concentration of both mono-and binuclear species in the conditions reported in the captions of Figure 1a–d. Figure 2 depicts the trend of mono-and binuclear complexes as we go from a solution with a small excess of ligand (Figure 2b) to a solution with an excess of metal ion (Figure 2d).

What catches the eye immediately is that the homobinuclear species have a relevant weight (25%) at physiological pH values even when Cu^2+^ and Ctr1_(1-14)_ are employed in a 1.2/1 ratio (Figure 2b), while they prevail when the metal ion exceeds the transporter (Figure 2d).

Mononuclear complex species are mainly formed with small Cu^2+^/Ctr1_14_ ratios (Figure 1a,b), while binuclear species prevail as the Cu^2+^/Ctr1_14_ ratio increases (Figure 1c,d and Figure 2c,d). Although small spectroscopic differences have been detected between the group of species that is detected in acidic conditions (CuLH_2_, CuLH) and the one that is formed in the alkaline region (CuL, CuLH_-1_, CuLH_-2_), all of these species have been reported to have a 4N coordination environment and to be characterized by the Cu^2+^-ATCUN motif. We will restrict the discussion to binuclear species (Table 2) since mononuclear species have been discussed by others [17].

The potentiometric analysis evidences the formation of a binuclear species, formally [Cu_2_L], which would be better represented as [Cu_2_(HL)H_-1_], since tyrosine is not deprotonated at these pH values. The deconvoluted UV-vis bands suggest that the formation of two different Cu^2+^ binding sites have different coordination environments. The UV-vis band is broad due to the contribution of two distinct absorption maxima quite away from each other. The first maximum is centered at 523 nm and is diagnostic of an ATCUN binding characterized by a {N_NH2_, 2N^-^, N_Im_} coordination environment localized in the MDH domain of the Ctr1_(1-14)_. The second maximum is centered at 704 nm and a value typically observed for a 1N1O coordination mode in which, beside an imidazole nitrogen of a histidine residue (His5 or His6), a carbonyl or carboxyl oxygen is bound to the metal ion [39]. The presence of two distinct binding sites for Cu^2+^ is corroborated by the spectroscopic values obtained through the deconvolution of CD spectra. The negative band at 272 nm provides evidence for the concurrent contributions of the N_Im_ → Cu^2+^ and N_NH2_ → Cu^2+^ CT bands. The presence of an ATCUN coordination motif and a second binding site for Cu^2+^ causes an increase of the ∆ε value of the N_Im_ → Cu^2+^ CT band as well as an inversion of the sign of the dichroic signal. The increase of the ∆ε value of the N^-^_amide_ → Cu^2+^ CT band at 314 nm supports the coordination of a second nitrogen atom of a deprotonated amide in the ATCUN site, the typical spectroscopic parameters of which [40] are summarized in Table 3.

The species resulting from the deprotonation of [Cu_2_L], formally [Cu_2_LH_-1_] but basically [Cu_2_(HL)H_-2_], reaches the formation maximum at pH 6.2; its pK value (pK_21-1_ = log β_21-1_ − log β_210_ = 5.91) suggests the involvement of a further imidazole nitrogen of a histidine residue (His5 or His6) in the second binding site. This pK value is very close to that found by La Mendola et al. [41] for Cu^2+^ bound to two adjacent histidine residues in a peptide with a sequence similar to that of Ctr1_(1-14)_.

At physiological pH values (Figure 1d), [Cu_2_LH_-2_] (equivalent to [Cu_2_(HL)H_-3_]) is the main species. Its pK value (pK_21-2_ = log β_21-2_ − log β_21-1_ = 6.55) indicates the deprotonation of a further amide nitrogen atom [42]. The spectroscopic data strongly suggest that such a deprotonation increases the copper affinity of the second binding site. The PeakFit analysis reveals two bands with λ_max_ at 522 nm (ACTUN site) and 590 nm (second binding site); likely, the second Cu^2+^ binding site has a 3N1O (2N_Im_, N^-^, O_COO_-) coordination environment in agreement with the literature data for similar Cu^2+^ peptide sequences [41,42]. In addition, an increase in the ∆ε value of the N^-^_amide_ → Cu^2+^ CT band (314 nm) of [Cu_2_LH_-2_] further supports the Cu^2+^ binding mode suggested above, which impacts on the overall conformation of the system. In [Cu_2_LH_-2_] the [Cu_2_L] CD band at 272 nm splits in a N_Im_ → Cu^2+^ CT band (λ_max_ = 260 nm) with a positive ∆ε value and a negative N_NH2_ → Cu^2+^ CT band at 281 nm.

A further pH increase causes the formation of [Cu_2_LH_-3_] (basically [Cu_2_(HL)H_-4_]); its pK value (pK_21-3_ = logβ_21-3_ − logβ_21-2_ = 7.80) is consistent with a 4N {2N_Im_, 2N^-^} [41] coordination mode resulting from the involvement of an additional deprotonated amide nitrogen. For [Cu_2_LH_-3_], the overall UV-vis band at 545 nm of the deconvoluted (HyperQuad) UV-vis spectrum has a symmetrical shape. The PeakFit analysis indicates a first band with λ_max_ at 522 nm for the ACTUN motif and a second band with λ_max_ at 560 nm for the 4N {2N_Im_, 2N^-^} coordination environment of the second binding site (Table 2). Both a further increase in ∆ε value and a slight red shift of the N^-^_amide_ → Cu^2+^ CT band centered around 320 nm, obtained by deconvoluting the [Cu_2_LH_-3_] CD spectrum, suggest a Cu^2+^ new coordination environment for the second binding site.

Between pH 9 and 11, the OH group of tyrosine deprotonates results in the formation of [Cu_2_LH_-4_] and [Cu_2_LH_-5_], in which both copper(II) ions are coordinated to the Ctr1_(1-14)_, each through four nitrogen atoms. The pK value for [Cu_2_LH_-4_] (pK_21-4_ = log β_21-4_ − log β_21-3_ = 8.72) is consistent with the coordination of a further amide nitrogen with a 4N {N_Im_, 3N^-^} coordination environment. The deconvoluted UV-vis spectrum for [Cu_2_LH_-4_], while maintaining the same shape found for the previous complex species, is characterized by both a further slight blue shift and an increase in ε_max_. The overall UV-vis band is centered at 535 nm; the PeakFit analysis evidences both the band ascribed to the ATCUN motif (λ_max_ at 522 nm) and the blue-shifted band related to second metal binding site (λ_max_ at 540 nm). No further changes in the ∆ε value for the N_NH2_ → Cu^2+^ CT band at 284 are observed in the deconvoluted CD spectrum for [Cu_2_LH_-4_], whereas the intensity of the N^-^_amide_ → Cu^2+^ CT band centered around 320 nm increases. The pK value of [Cu_2_LH_-5_] (pK_21-5_ = log β_21-5_ − log β_21-4_ = 10.72) is consistent with the deprotonation of the phenolic group of the tyrosine residue, which takes no part in the coordination to the Cu^2+^. The similarity between the [Cu_2_LH_-5_] and [Cu_2_LH_-4_] spectroscopical parameters calculated strongly supports the idea that the deprotonation of [Cu_2_LH_-4_] involves no change in the metal centers coordination environment.

Ctr1_(1-14)_ has been proposed as a model of the extracellular acquisition of Cu^2+^ ion from HSA, although it is a short domain of the trimeric protein; Cu^2+^ bound to the ATCUN motif would be reduced by ascorbic acid, and the resulting Cu^+^ would be transferred to the adjacent histidine motif encompassing His5 and His6 that, in turn, would stabilize Cu^+^ [23,27,28]. Such a pathway would allow for the overcoming of the issue related to the well-known inability of Cu^2+^ coordinated to 4N ATCUN motif to oxidize ascorbic acid [35,36]. Instead, our findings demonstrate that Ctr1_(1-14)_ can bind two equivalents of Cu^2+^ and thus suggest an exciting alternative to the reduction mediated by the ATCUN motif, that is, the involvement of a second binding site. Interestingly, this second Cu^2+^ binding site detected in the present study is characterized by a 4N (2N_Im_, 2N^-^) coordination environment having a lesser metal affinity constant than that related to the Cu^2+^ affinity for the ATCUN motif and consequently stands as a valid candidate for Cu^2+^ reduction.

### 2.2. Cu^2+^ Complexes with Ctr1_(1-14)_ Oxidize Ascorbic Acid When Binuclear Species Form

The oxidation kinetics of ascorbate has been extensively investigated employing both free Cu^2+^ and Cu^2+^ complexes [43]; these data show that the different Cu^2+^ binding environments drive the kinetics and mechanisms of the ascorbate oxidation. Some studies report that different Cu^2+^ binding domains in the same protein or polypeptides display different ascorbate oxidation abilities [40,44].

Specifically, copper(II) complexes with peptides encompassing the ATCUN motif (DAHK) in human serum albumin, Aβ (both Aβ_(4-16)_ and Aβ_(12-20)_) and Ctr1 (MDH), do not significantly oxidize ascorbate, suggesting that the metal 4N {NH_2_, N_Im_, 2N^-^} coordination mode is responsible for the lack of oxidation. Conversely, it has also been suggested that the presence of a His-His motif adjacent to the ATCUN site in Ctr1_(1-14)_ helps to obtain the Cu^+^ species in the presence of ascorbate thanks to the binding of the reduced metal ion to the adjacent histidines of the His-His motif [23].

**Figure 3:**
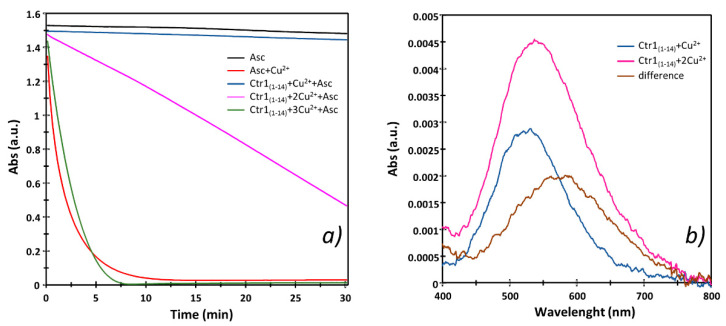
(**a**) Ascorbate-dependent reduction of Cu^2+^ in the presence or absence of Ctr1_(1-14)_ at different metal/peptide ratios. Peptide (25 μM) and ascorbate (100 μM) solutions in MOPS (10 mM) at pH 7.2 were monitored for 30 min by UV-vis spectra at 265 nm. Change of ascorbate absorbance band: ascorbate without (black line) and with Cu^2+^ (red line), 1:1 Cu^2+^/Ctr1_(1-14)_ ratio (blue), 2:1 Cu^2+^/Ctr1_(1-14)_ ratio (magenta), 3:1 Cu^2+^/Ctr1_(1-14)_ ratio (green). (**b**) UV-vis spectra of Cu^2+^-Ctr1_(1-14)_ system carried out in the same experimental condition of Cu^2+^ ascorbate-dependent reduction; the brown line represents the difference spectra.

**Figure 4:**
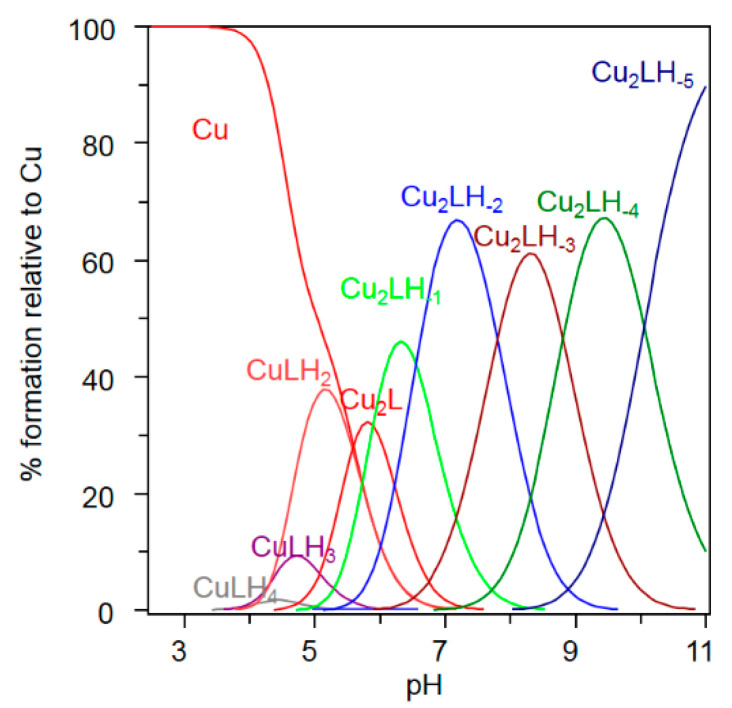
Species distribution for the Cu^2+^-Ctr1_(1-14)_ system. C_Ctr1_ = 2.5 × 10^−5^ mol dm^−3^; Cu^2+^/Ctr1_(1-14)_ concentration ratio is 1.9.

In order to compare the oxidation ability of the Cu^2+^ bound to the Ctr1_(1-14)_ ATCUN and secondary binding site, we measured the changes in the rates and levels of ascorbate consumption with changes of Ctr1_(1-14)_/Cu^2+^ ratios. Figure 3a shows that the addition of one equivalent of Cu^2+^ to the buffer causes the largest and fastest ascorbate consumption (red line). The addition of one equivalent of Ctr1_(1-14)_ to a buffered Cu^2+^ solution inhibits the Cu^2+^-mediated ascorbic acid oxidation (blue line) due to the formation of copper(II) ion complex with the MDH sequence of the N-terminus (ATCUN motif). Increasing the metal to peptide ratio, that is, favoring binuclear species formation (Figure 4), causes an increase in the kinetics of ascorbate consumption (magenta line) that, however, is not comparable to that measured for a buffered solution containing Cu^2+^ only (red line). Noteworthy is the fact that, if we push the metal/ligand ratio further up, we detect a fast ascorbate consumption (green line) similar to that found when one equivalent of only Cu^2+^ is added to the buffer (red line). This indicates that the excess Cu^2+^ that is no longer complexed by Ctr1_(1-14)_ causes a reduction of ascorbate comparable to that detected for the solution containing no ligand.

Figure 3b depicts the UV-vis spectra for the Cu^2+^-Ctr1_(1-14)_ system obtained with a 1:1 (blue line) and 2:1 (magenta line) Cu^2+^/Ctr1_(1-14)_ ratio in the same experimental conditions employed for the Cu^2+^ ascorbate-dependent reduction. This figure clearly shows that λ_max_ shifts towards greater wavelenghts as binuclear species prevail (Figure 4). The difference spectrum (brown line), obtained by subtracting the spectral contribution characteristic of the ATCUN binding site (blue line) from the spectrum obtained with a greater Cu^2+^/Ctr1_(1-14)_ ratio (magenta line), gives an idea of the characteristics of the Cu^2+^ second binding site, further indicating that such a binding site is weaker than the ATCUN motif. 

The above results support the idea that it is just the Cu^2+^ bound to the second site containing the His-His motif that is reduced by ascorbate. This changes the previous reduction model according to which Cu^2+^ coordinated to the ATCUN binding site would first be reduced by ascorbate and the resulting Cu^+^ would then be transferred to the His-His diade that is able to stabilize the reduced state of the metal ion [23,27,28,34]. Noteworthy is the fact that the literature data reveal that our model for the reduction of the Cu^2+^-Ctr1_(1-14)_ has been also suggested for HSA that contains an unspecific second metal binding domain [44]. This additional coordination site, characterized by an affinity to Cu^2+^ lower than that of the Cu^2+^ binding to the ATCUN motif of the protein, is thought to be responsible for ascorbate oxidation. In addition, a second binding site (sometimes termed ‘the secondary site’) in which Cu^2+^ is coordinated to His13 and His14 has been evidenced for Aβ_(4-16)_; as found for the second site HSA and Ctr1_(1-14)_ that binds Cu^2+^, this non-ATCUN Aβ-Cu^2+^ species has a micromolar affinity constant and oxidizes ascorbate [45].

### 2.3. Ag^+^ Forms Mononuclear Complex with Ctr1_(1-14)_

Cu^+^ binding affinity to the Ctr1 N-terminus has been studied using peptides with different sequences as well as different experimental approaches [23,30,33,34]. Initially, the Met-rich motif of yCtr1 was investigated [25]; the study was then extended to hCtr1 that also contains a His-rich sequence [23]. For the latter system, the conditional stability constants were determined by competition between Cu^+^ bound to Ctr1_(1-14)_ copper(I) complexes and bicinchoninic acid, the affinity constants of which are precisely known; experiments were also extended to polypeptides encompassing additional His and Met motifs. These experiments were carried out in anaerobic conditions to avoid the oxidation of [Cu(MeCN)_4_]PF_6_. A narrow metal to peptide ratio was explored in these competition experiments; the peculiar experimental conditions employed might be the cause of some contrasting data.

**Figure 5:**
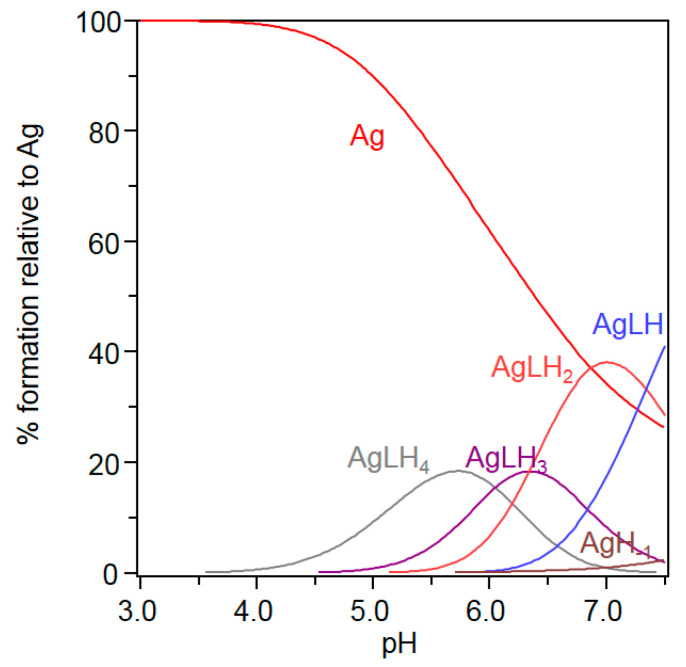
Species distribution of Ag^+^ complexes with Ctr1_(1-14)_ (L). Ag^+^/Ctr1_(1-14)_ = 0.9; the analytical concentration of Ctr1_(1-14)_ is 5 × 10^−4^ mol dm^−3^.

Since Cu^+^ cannot be studied straightforwardly in aqueous solutions and Ag^+^ is considered a good surrogate for monovalent copper [13,20], we investigated by potentiometry different Ag^+^/Ctr1_(1-14)_ ratios (1:1, 1:2 and 2:1) to determine the binding constants and to obtain the species distribution of Ag^+^ complexes with Ctr1_(1-14)_. The treatment of the potentiometric data, which also included Ag^+^ hydrolytic species [46], invariably converged towards the set of species listed in Table 4; the distribution diagram of these species is shown in Figure 5. 

[AgLH_4_] starts to form around pH 4 and reaches its maximum percentage of formation close to pH 6, where the carboxylic groups are deprotonated; log K* (log K*_114_ = log β_114_ − log β_014_ = 33.45 − 30.47 = 2.98) value suggests that at least one imidazole nitrogen of a deprotonated His residue is involved in the binding, while the remaining histidine moieties and the amino and tyrosine residues are protonated. As pH increases, [AgLH_4_] loses a proton yielding [AgLH_3_], the log K* of which (log K*_113_ = log β_113_ − log β_013_ = 27.40 − 24.18 = 3.22) is consistent with a 2N {2N_Im_} binding environment resulting from the coordination of an additional imidazole nitrogen of a second deprotonated His residue. The log K* (log K*_112_ = log β_112_ − log β_012_ = 21.05 − 17.27 = 3.78) of the species that prevails at physiological pH ([AgLH_2_]) is consistent with a third deprotonated His.

In this context, in an effort to understand whether the His or Met residue is involved in the binding, a systematic study of metal complexes with mutated Ctr1_(1-14)_, in which the His and Met residues have been replaced by alanine or norleucine residues, has been carried out [23]. The affinity constant values reported by this study evidence that the simultaneous substitution of His5 and His6 as well as the replacement of His3 result in a significant decrease of the log K found for the un-mutated peptide, suggesting a possible involvement of the histidine residue of the ATCUN motif in Cu^+^ binding. On the other hand, the replacement of all Met residues also affects the metal affinity, though the decrease of log K is smaller than that displayed by the His mutated peptides. His3 involvement in Cu^+^ binding to monomeric MDHSHH and its trimeric derivative is also supported by an NMR investigation showing that Met1 is also involved in Cu^+^ coordination to these Ctr1_(1-14)_ metal binding models [27]. In addition, NMR findings show that in the wild-type peptide in the presence of Cu^+^, the most significant chemical shifts are detected for the His and Met residues, suggesting that His3, Met7, Met9 and Met12 are the most relevant residues for Cu^+^ binding [29]. However, converging evidence from XAS, computational and NMR results supports the involvement of His5 and His6 in Cu^+^-Ctr1_(1-14)_ complexes with a {2N_im_, 1O, 1S} coordination mode [27,47].

Above the physiological pH range, only one Ag^+^ complex species ([AgLH]) that gradually makes way for Ag^+^ hydroxylated species is detected; the precipitation of this latter species prevents a reliable investigation of the effect resulting from the (possible) deprotonation of the tyrosine OH group. AgLH formation is associated with a pK value (pK_111_ = log β_112_ − log β_111_ = 7.33) that recalls the value of the proton dissociation constant of the terminal α-amino group, which is not able to bind silver ion. 

### 2.4. The Magnetic Parameters Values Obtained by Electro Spin Resonance (ESR) Measurements Indicate That the Two Binding Sites of Ctr1_(1-14)_ Experience a Different Coordination Environment

**Figure 6:**
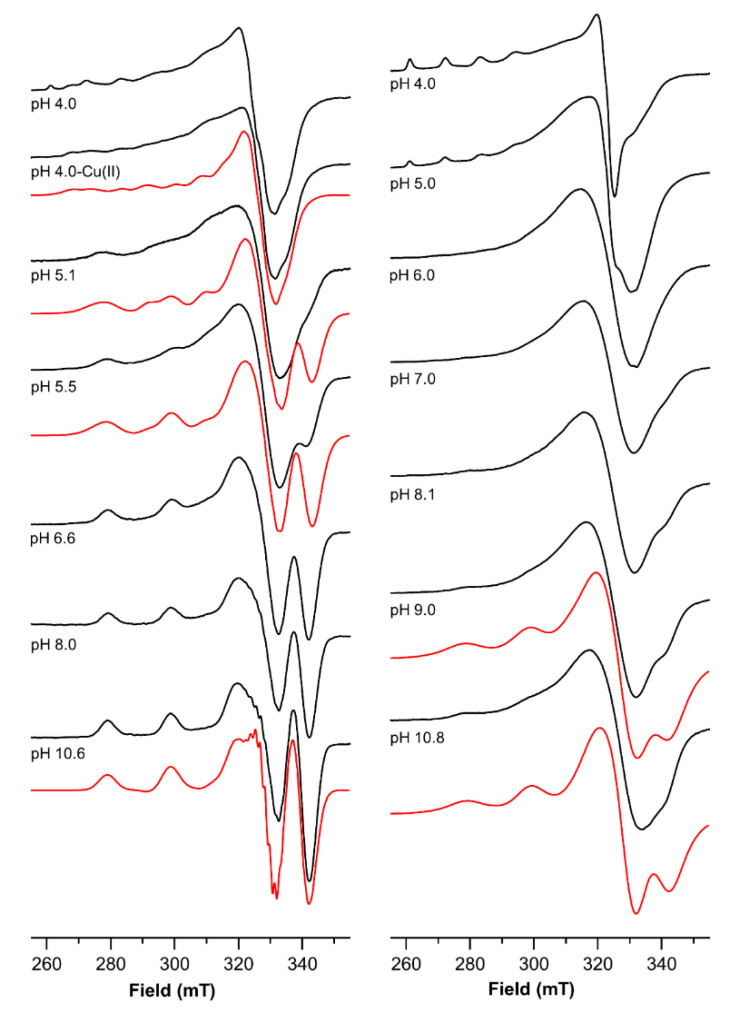
ESR spectra (150 K) of the complex species of Ctr1_(1-14)_, with Cu^2+^ as function of the pH and the ligand-to-metal ratio (left panel, L:Cu = 1:1; right panel, L:Cu = 1:2). Computed spectra are shown in red.

Studies of the native peptide as well as shorter fragments or mutated peptides have reported the magnetic parameters of the complex of Cu^2+^ with the N-terminus of Ctr1_(1-14)_ involving an ATCUN motif [17,27,28,29,32]. It is generally accepted that the metal binding to the N-terminal domain of Ctr1 is characterized by a 4N (an amino nitrogen, an imidazole nitrogen and two deprotonated amide nitrogens) coordination mode. The g_‖_ and A_‖_ values obtained for the Cu^2+^ mononuclear complex with a 0.9 metal-to-ligand ratio were used for the attribution of the parameters of the analogous 4N complex found for the Cu^2+^ complexes with Ctr1_(1-14)_ detected with a 1.8 metal to ligand ratio over a large pH range. For the mononuclear complex (0.9 metal-to-ligand ratio), about one equivalent of ^63^Cu^2+^ was added to a peptide solution adjusting the pH value, which was then adjusted to 4.0. The ESR spectrum run on this frozen solution shows at least two patterns of signals beside those due to Cu^2+^ hexaaquo ion (Figure 6, Table 5); for a better visualization of the pattern of the complex species, the features of the hexaaquo ion (spectrum obtained at pH 3 not shown) were subtracted from the overall spectrum. The Hamiltonian parameters of the species were obtained directly from the resulting spectrum and used as initial values for the simulation of the subtracted spectrum. The simulation yields the following values: g_‖_ = 2.307, A_‖_ = 172 × 10^−4^ cm^−1^ and g_‖_ = 2.245, A_‖_ = 178 × 10^−4^ cm^−1^. As the pH is raised to 4.5, the spectral contribution of latter chromophore predominates though the ESR spectrum does not change significantly. A further pH increase (5.0–5.5) causes a sizable change in the ESR spectra. The Hamiltonian parameters obtained by simulating the spectra collected over this pH range are: g_‖_ = 2.177, A_‖_ = 205 × 10^−4^ cm^−1^ and g_‖_ = 2.230, A_‖_ = 185 × 10^−4^ cm^−1^. The first set of parameters is typically obtained for ATCUN Cu^2+^ complexes [37]. The second set of Hamiltonian parameters indicates the formation of a different second coordination site. This site can be assigned to the coordination of a histidinic imidazole nitrogen and two deprotonated amide nitrogen atoms [41].

Above pH = 6.6, only the ATCUN features are detected in the ESR spectra as they are also found by other groups [37]; however, the Hamiltonian parameters do not significantly change as we move towards more alkaline regions.

The ESR spectrum runs on a solution of Ctr1_(1-14)_ containing 1.8 equivalents of ^63^Cu^2+^ at pH = 4 and mainly shows the features of the hexaaquo metal ion. Over the pH range 5–7, despite the strong decrease of the signals of hexaaquo copper ion that progressively fade to disappear above pH 6, the spectra are almost featureless in the parallel part, while they show a very broad signal in the perpendicular part. The broadening likely results from the formation of binuclear complex species characterized by similar coordination environments. [Cu_2_L], [Cu_2_LH_-1_] and [Cu_2_LH_-2_] are present in this pH range. In these species, one Cu^2+^ has an ATCUN {NH_2_, N_Im_, 2N^-^} coordination mode, while the second metal ion is gradually anchored to His5 or/and His6 and also involves a carboxylate and a deprotonated amide nitrogen. Thus, the second coordination center has a {N_Im_, O_COO-_}, {2N_Im_, O_COO_-} or {2N_Im_, N^-^, O_COO_-} coordination mode as the ligand is progressively deprotonated.

Since the magnetic interaction of the two copper ions strongly depends on their mutual distance, [Cu_2_LH_-2_] and [Cu_2_LH_-3_] were modeled by HyperChem (HyperChem, Hypercube, 2000) [48,49] to have an estimate of the Cu-Cu distance. The carboxylate oxygen is most probably provided by the Asp2 residue, and this would bring the two copper ions into close proximity (i.e., about 5.7 Å) in the frozen solution, thus causing the broadening of the ESR signal due to the dipolar interaction [50]. Aggregation effects might also be involved in the severe broadening occurring at low temperatures in the ESR spectra, as observed by Shenberger et al. [29].

As we move to more alkaline regions, the further deprotonation leads to a 4N {2N_Im_, 2N^-^} coordination mode, with a loss of the coordinated Asp. The two Cu^2+^ ions move apart (i.e., about 7.3 Å), thus weakening the dipolar interaction. Consequently, above pH 8, the parallel features of the ESR spectra become visible, although some broadening still persists. A signal at 340 mT is also detected, which may be ascribed to the ATCUN spectrum recorded in alkaline regions for solutions containing one Cu^2+^ equivalent (Figure 6). The magnetic parameters in alkaline regions were obtained by simulation using the parameters of the {NH_2_, N_Im_, 2N^-^} and the {2N_Im_, 2N^-^} coordination modes, i.e., g_∥_ = 2.181, A_∥_ = 205 × 10^−4^ cm^−1^ and g_∥_ = 2.210, A_∥_ = 185 × 10^−4^ cm^−1^, respectively, and introducing in the simulation larger linewidth values, since some of the spectra show line broadening.

### 2.5. Immunoreactivity of Ctr1 Peptides of Different Length with Anti-Ctr1 Extracellular Domain Antibody

The 2N1O1S coordination mode of the Cu^+^ complex with Ctr1_(1-14)_ is functional both to the N-terminus reductase ability and to the transport of metal ion via the ectodomain C-terminus region responsible for cytoplasm metal uptake. The labile complex formation would favour Cu^+^ exchanges involving a series of labile sites down a concentration gradient and/or a thermodynamic stability gradient, as suggested by Pushie et al. [27]. A recent report emphasizes the role played by Cu^+^ binding to hCtr1_(1–46)_ in the peptide folding that is in turn responsible for its interactions with cell membranes; noteworthy is the fact that apo-hCtr1_(1–46)_ does not interact with the membrane. The conformation change has been ascribed to the metal ion coordination to some residues of the first and second His-rich and Met-rich domain of Ctr1_(1-46)_, which encompasses different copper binding sites such as His23–His6, His6–His33 and His23–Met7 [33].

Inspired by these findings and by the models proposed so far, we have performed preliminary assays to compare the molecular recognition by an anti-Ctr1 extracellular antibody of the different ectodomains present in Ctr1_(1-14)_ and a longer peptide Ctr1_(1-25)_ in the absence and presence of copper to gain further information on the interaction with the membrane.

**Figure 7:**
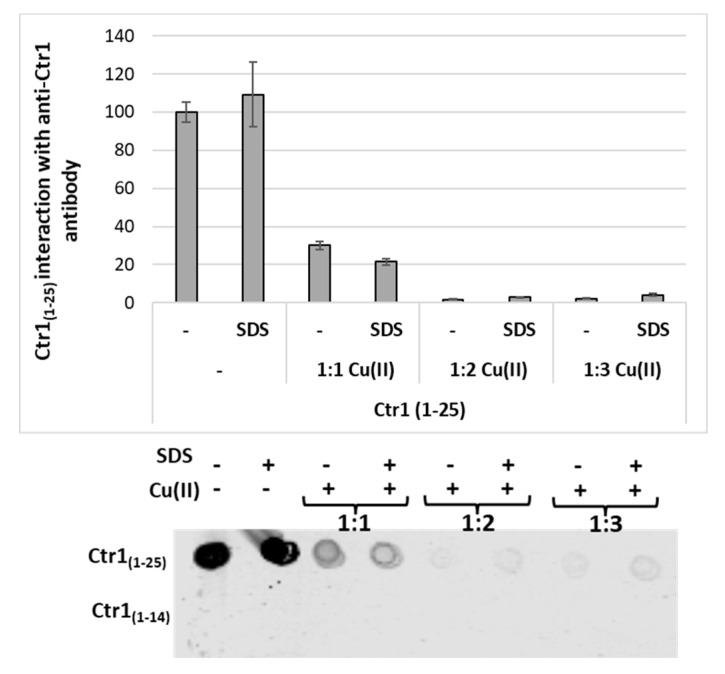
Immunoblots of Ctr1_(1-14)_ and Ctr1_(1-25)_ peptides (1 mM) pre-incubated with different concentrations of copper (L/Cu^2+^ ratios 1:1, 1:2, 1:3) with or without 1% SDS. The membrane was incubated with anti-Ctr1 extracellular domain antibody.

The dot blot analysis shows that the antibody recognizes the generic epitope associated with Ctr1_(1-25)_, while it does not recognize the shorter Ctr1_(1-14)_ (Figure 7). Both peptides (1 mM in phosphate buffer) were also pre-incubated for 15 min with copper in different ratios (L/Cu^2+^ 1:1, 1:2 and 1:3). The dot blot analysis shows a decrease of antibody affinity for Ctr1_(1-25)_ following pre-incubation, with copper in the samples containing the metal ion at a ratio of 1:1 and no interactions with antibodies following incubation with higher metal-to-ligand ratios (L/Cu^2+^ 1:2 and 1:3). The incubation was carried out in the presence of 1% SDS in order to verify the influence of the native peptide conformation on antibody binding. Noteworthy is the fact that the antibody does not recognize Ctr1_(1-14)_ when the peptide is used without adding copper nor when it is preincubated with copper.

Figure 7 shows that (i) the interaction of Ctr1 peptides changes with the length of the peptide sequence, and (ii) Cu^2+^ binding to the N-terminal metal binding domain of Ctr1_(1-25)_ peptide likely induces a conformational change that in turn inhibits the interaction with the anti-Ctr1 extracellular antibody.

Our results evidence that the choice of peptide length is crucial to obtaining a model that appropriately represents Ctr1. hCtr1 N-terminal extracellular domain contains two Met-rich as well as two His-rich motifs involved in the coordination of copper ions, which have been extensively studied by using different model peptides (full N-terminal domain, Ctr1_(1-14)_, Ctr1_(1-46)_) [23,30]. Future and deeper investigation of a longer peptide model of Ctr1 (namely, Ctr1_(1-25)_) will hopefully help to better clarify how the extracellular localization and the copper binding capacity of the hCTR1 N-terminus allow the protein to recruit copper ions from the extracellular environment.

### 2.6. Concluding Remarks

hCTR1 ectodomain has two Met-rich motifs, i.e., the two ^7^MXMXXM and ^41^MMMXM sequences, and two His-rich motifs arranged as ^1^MDHXHH and ^22^HHH. Initially, the findings highlighted that removing the Met motifs nullified the CTR1-assisted uptake of copper [51]. Later on, studies on a short peptide fragment (Ctr1_(1-14))_ [23] as well as on longer peptides (Ctr1_(1-46)_ [33] and Ctr1_(1-55)_ [30]) emphasized the role of the histidine residues of both the first and second His-rich motifs in the binding of the metal ion. The incubation of long peptides with different Cu^2+^/Ctr1 ratios [33] showed that the extracellular part of hCtr1 can bind up to three Cu^2+^ and six Cu^+^, while Ctr1_(1-14)_ binds one Cu^2+^ through the MDH sequence; the study also showed that His5 and His6 residues contribute to the reduction of Cu^2+^ in the presence of ascorbate [34].

Our study unveils the formation of Cu^2+^ binuclear species, the second binding site of which involves the two imidazole nitrogen atoms of the His5 and His6 residues. The second binding site is characterized by an affinity lower than that of the first Cu^2+^ bound to the Ctr1_(1-14)_ ATCUN motif and is also responsible for ascorbate oxidation. These new results contribute to a better description of the features of hCtr1_(1-14)_ as a reductase model of the extracellular N-terminus copper binding portion. In light of the role played by the second His-rich motif, we are currently investigating a longer peptide (Ctr1_(1-25)_) that encompasses the ^22^HHH sequence by using the same approach employed in the present study. 

## 3. Materials and Methods

### 3.1. Chemicals

Ctr1_(1-14)_ and Ctr1_(1-25)_ were supplied by Caslo Aps, Lyngby, Denmark. All other chemicals (Sigma-Aldrich -Munich, Germany) were of the highest available grade and were employed without further purification.

Since, as also found for some analogous shorter peptides [32], Ctr1_(1-14)_ may contain water and the accurate determination of the thermodynamic parameters requires concentrations to be precisely known, the water content of stock solutions was checked by potentiometric (protonation) titrations, as routinely done in our group for newly synthesized ligand as well as for ligands that tend to absorb water from the surrounding environment [52,53,54]. The water content was double checked by thermogravimetric analysis (TGA). The samples (1–2 mg) were analyzed (TA Instruments Q500, New Castle, DE, USA) under a nitrogen flow (60 mL⋅min^−1^) using a heating rate of 5 °C/min and 10 °C/min in 30-200 and 200–800 °C, respectively (sensitivity = 2.5 × 10^−6^ g). The TGA analysis shows that, within the experimental error, the ligand contains the same amount of water (*ca*. 25%) determined by potentiometric titrations and undergoes complete decomposition in the 200–300 °C range; no further weight loss was detected up to 800 °C, indicating that the ligand is free from inorganic impurities. The above figure was considered to have the correct analytical concentration for potentiometric as well as spectroscopic experiments.

### 3.2. Electron Spin Resonance Spectroscopy (ESR)

A Bruker CW-ESR spectrometer (Elexsys E500, Bruker, Karlsruhe, Germany) driven by a PC running the XEpr program and equipped with a Super-X microwave bridge (ER 049X) operating at 9.3–9.5 GHz and a SHQE probe head were used throughout the work. All frozen solution ESR spectra of Cu^2+^ complexes were recorded at 150 K by means of an ER4131VT variable temperature unit. A small amount of methanol (up to 10%) was added to the Cu^2+^-Ctr1 samples in order to increase spectral resolution. Parallel spin Hamiltonian parameters were taken directly from the experimental spectra and were always calculated from the 2nd and 3rd line to get rid of errors coming from second order effects [55]. The resolution of the ESR spectra [56] was improved by using isotopically pure ^63^Cu(NO_3_)_2_ (0.05 M). In order to achieve a better determination of the magnetic parameters, some of the experimental spectra were simulated by the program Monoclin [57,58], which is able to discriminate one or more species [57]. Instrumental settings of frozen solution ESR spectra were as follows: number of scans, 4; microwave frequency, 9.425–9.429 GHz; modulation frequency, 100 kHz; modulation amplitude, 0.7 mT; time constant, 163 ms; sweep time, 2.8 min; microwave power, 20 mW; receiver gain, 60 dB.

### 3.3. Potentiometric Titrations

Potentiometric experiments were performed using a Titrando 905 automatic titrator (Switzerland) equipped with a high alkalinity combined glass-Ag/AgCl electrode (Metrohm, Switzerland). The titration cell (2.5 mL) was thermostatted at 298.0 ± 0.2 K, and all solutions were kept under an atmosphere of argon. KOH solutions (0.1 M) were added through a Hamilton burette equipped with a 1 cm^3^ syringe. The ionic strength of all solutions was adjusted to 0.10 M (KNO_3_). In order to determine the stability constants, solutions of Cu^2+^ ion and Ctr1_(1-14)_ were titrated using 0.1 M potassium hydroxide. Ligand concentration ranged from 1.0 to 1.5 × 10^−3^ M. The Cu^2+^ to Ctr1_(1-14)_ concentration ratio covered a wide range spanning from 0.9 to 1.9. A minimum of three independent runs were performed to determine Cu^2+^ complexation constants. 

The initial pH was always adjusted to 2.4. To avoid systematic errors and check for reproducibility, the electromotive force (EMF) values of each experiment were taken at different time intervals. Hyperquad [59] and HySS [60] were used to calculate complexation constants from potentiometric data and species distribution diagrams, respectively. 

### 3.4. Ultraviolet-visible (UV-vis) Measurements

UV-vis spectra were recorded at 25 °C using an Agilent 8453 (Agilent Technologies, Santa Clara, CA, USA) or a Jasco V-670 (Jasco, Easton, MD, USA) spectrophotometer. The concentrations of the peptide and Cu^2+^ used to record absorption spectra were the same as those for the potentiometric titrations.

Combined spectroscopic and potentiometric metal-complex titrations were performed into a 3 mL quartz cuvette with a 1 cm path length to obtain the spectrum in the visible region at each pH value. These experiments were replicated at least three times for each copper-peptide system. Spectroscopic data were processed using Hyperquad [58]. UV-vis spectra were deconvoluted using PeakFit 4.0 (Jandel Scientific Software).

### 3.5. Circular Dichroism (CD) Experiments

CD spectra were obtained at 25 °C under a constant flow of nitrogen in the 280–800 nm range with a model 810 Jasco spectropolarimeter (Jasco, Easton, MD, USA) at a scan rate of 50 nm min^−1^, a resolution of 0.1 nm and a 1 cm path length. The spectra were recorded as an average of 3–5 scans. Calibration of the instrument was performed with a 0.06% aqueous solution of ammonium camphor sulfonate. The CD spectra of Cu^2+^ complexes as a function of the pH were obtained in both the 190–250 and 250–800 nm regions. All solutions were freshly prepared using twice distilled water. Cu^2+^ and peptide concentrations used for CD spectra in the visible region were identical to those used in the potentiometric titrations. Results are reported as Δε (molar dichroic coefficient) in M^−1^ cm^−1^.

### 3.6. Ultraviolet (UV) Determination of Ascorbate Consumption

Ascorbate consumption was monitored using a Jasco V-670 spectrophotometer. The intensity of the ascorbate (100 μM) absorption band at λ = 265 nm was monitored as a function of time in 10 mM MOPS (pH = 7.4). Cu^2+^ and Ctr1_(1-14)_ concentrations were 20 μM and 25 μM, respectively; a 1 cm path-length quartz cell (slit width = 2 nm) was employed throughout.

A stock solution (20 mM) of ascorbate was prepared in milli-Q water just before the experiment and was used immediately. Owing to the instability of ascorbate solutions, fresh solutions were prepared for each experiment. Cu(NO_3_)_2_ stock solutions were prepared and standardized with ethylenediaminetetraacetic acid, as reported elsewhere [61].

### 3.7. Dot Blot Assay 

Ctr1_(1-25)_ and Ctr1_(1-14)_ (1 mM) peptides were pre-incubated in 10 mM phosphate buffer, pH 7.0 with different concentrations of copper (ratios 1:1, 1:2, 1:3) with or without 1% SDS. In total, 2 μL of each sample were pipetted onto a Whatman nitrocellulose membrane (GE Healthcare, Pittsburgh, PA) which corresponds to 10 µg of Ctr1_(1-25)_ and Ctr1(_1-14_). After the last sample was deposited, membranes were allowed to air-dry. Non-specific binding was blocked by incubation with an Intercept Blocking Buffer (LI-COR Biosciences) for 1 h at room temperature (RT). Membranes were then incubated with anti-Ctr1 extracellular domain antibody (ab129067, dilution 1:2000) for 4 h at RT. After three 5-min washes in PBS-Tween 20, the membranes were incubated with goat anti-rabbit antibody labeled with IRDye 680 (1:26,000 dilution, LI-COR Biosciences) for 1 h at RT. The blots were then washed 3 times for 5 min each in PBS-Tween 20, and hybridization signals were detected with the Odyssey Infrared Imaging System (LI-COR Biosciences). Representative results from three independent experiments are shown.

## Figures and Tables

**Table 1:** Log β and pK values for Cu^2+^ complexes with Ctr1_(1-14)_ at I = 0.1 mol dm^−3^ (KNO_3_) and T = 298 K.

Species [Cu_p_L_q_H_r_]	log β ^a,b^	pK ^b^	log β ^c^	pK ^c^	Coordination Mode ^d^	
pqr						
114	35.07 (7)	-	35.37	-	—	
113	31.38 (3)	3.69	31.62	3.75	—	
112	26.82 (3)	4.56	27.04	4.58	—	
111	21.44 (4)	5.39	21.64	5.40	—	
110	15.21 (4)	6.23	15.37	6.27	—	
11-1	7.80 (5)	7.41	8.25	7.12	—	
11-2	−1.96 (6)	9.77	−1.74	9.99	—	
210	20.63 (3)	—	—	—	4N {NH_2_, 2N^-^, N_Im_}	1N1O {N_Im_, O_COO_^-^}
21-1	14.72 (3)	5.91	—	—	4N {NH_2_, 2N^-^, N_Im_}	2N1O {2N_Im_, O_COO_^-^}
21-2	8.37 (5)	6.34	—	—	4N {NH_2_, 2N^-^, N_Im_}	3N1O {2N_Im_, N^-^, O_COO_^-^}
21-3	0.85 (4)	7.53	—	—	4N {NH_2_, 2N^-^, N_Im_}	4N {2N_Im_, 2N^-^}
21-4	−7.87 (4)	8.71	—	—	4N {NH_2_, 3N^-^}	4N {N_Im_, 3N^-^}
21-5	−18.59 (4)	9.96	—	—	4N {NH_2_, 3N^-^}	4N {N_Im_, 3N^-^}

^a^ 3σ in parentheses; ^b^ This work; ^c^ Reference 17; ^d^ Proposed coordination modes for binuclear species discussed in the body text.

**Table 2:** Spectroscopic parameters obtained from the titration of the Cu^2+^-Ctr1_(1-14)_.

Species [Cu_p_L_q_H_r_]	UV-vis λ, nm (ε, M^−1^ cm^−1^)		CD λ, nm (∆ε, M^−1^ cm^−1^)	g_∥_	A_∥_ × 10^4^ cm^−1^
pqr					
210	523 (130)	704 (35)	272 (−1.36), 314 (+0.39), 490 (+0.34), 580 (−0.56)	—	—
21-1	—	—	—	—	—
21-2	522 (130)	590 (90)	260 (+0.94), 281 (−0.75), 314 (+0.76), 492 (+0.97), 580 (−0.90)	—	—
21-3	522 (130)	560 (110)	260 (+0.91), 281 (−0.91), 323 (+1.03), 492 (+1.18), 580 (−0.94)	2.177 2.230	205 185
21-4	522 (130)	540 (125)	260 (+0.71), 284 (−1.33), 323 (+1.16), 492 (+1.24), 580 (−1.01)	2.181	203
21-5	522 (130)	540 (130)	260 (+0.69), 284 (−1.30), 320 (+1.12), 492 (+1.10), 580 (−1.01)	2.181	203

3σ in parentheses.

**Table 3:** Typical spectroscopic features of the ATCUN XZH motif of Cu^2+^ complexes *.

Motif	UV-vis	CD		EPR	
	λ_max_	d-d Transitions	CT	g_||_	A_||_ (10^4^ × cm^−1^)
XZH	~520 nm	~480 nm ~560 nm	~310 nm	~2.190	~200

* Reference [40].

**Table 4:** Log β values obtained for the Ag^+^-Ctr1_(1-14)_ system at I = 0.1 mol dm^−3^ (KNO_3_) and T = 298 K.

Species [Ag_p_L_q_H_r_]	log β ^a^	pK	log K*	Coordination Mode
pqr				
114	33.45 (3)	—	2.98	1N {N_Im_/1N1O1S}
113	27.40 (4)	6.05	3.22	2N {2 N_Im_/2N1O1S}
112	21.05 (3)	6.35	3.78	2N {2 N_Im_/2N1OS}
111	13.72 (2)	7.33	4.28	2N {2 N_Im_/2N1OS}
10-1	−8.56 ^b^	—	—	—

^a^ 3σ in parentheses; ^b^ Reference [46].

**Table 5:** ESR parallel Hamiltonian parameters of Cu^2+^ with Ctr1_(1-14)_ at different metal-to-ligand ratios as a function of the pH.

L:Cu 1:1			L:Cu 1:2		
pH	g_∥_	A_∥_ × 10^4^ cm^−1^	pH	g_∥_	A_∥_ × 10^4^ cm^−1^
4.0	2.245(3)	178(3)	4.0	2.424(3)	124(3)
	2.307(3)	172(3)		lbr	lbr
	2.424(2)	124(2)			
5.1	2.177(2)	205(3)	5.0	2.424(3)	124(3)
	2.230(3)	185(3)		lbr	lbr
5.5	2.177(2)	205(3)			
	2.230(3)	185(3)			
			6.1	lbr	lbr
6.6	2.177(2)	205(3)	7.0	2.181(5)	203(5)
				lbr	lbr
8.0	2.181(3)	203(3)	8.1	2.181(5)	203(5)
				lbr	lbr
			9.0	2.181(5)	205(5)
				2.210(5)	185(5)
10.6	2.181(3)	203(3)	10.8	2.181(5)	205(5)
				2.210(5)	185(5)

lbr—line broadening.

## Data Availability

Not applicable.

## Supplementary Materials

The following supporting information can be downloaded at: https://www.mdpi.com/article/10.3390/ijms23062929/s1.
