# Supplementary material for: A Deeper Insight in Metal Binding to the hCtr1 N-terminus Fragment: Affinity, Speciation and Binding Mode of Binuclear Cu2+ and Mononuclear Ag+ Complex Species
Source: Int J Mol Sci. 2022 Mar 8;23(6):2929. doi: 10.3390/ijms23062929 (PMC8953729; doi:10.3390/ijms23062929)
Supplement: Supplementary file 1 [file ijms-23-02929-s001.zip › A deeper insight in metal binding -supplementary.pdf]

Supplementary Materials

A deeper insight in metal binding to the hCtr1 N-terminus fragment: affinity, speciation and binding mode of binuclear Cu^{2+} and mononuclear Ag^{+} complex species

Antonio Magrì[1,#], Giovanni Tabbì[1,#], Irina Naletova[1,2], Francesco Attanasio[1,*], Giuseppe Arena[3] and Enrico Rizzarelli[1,2,3,*]

[1] Institute of Crystallography, National Council of Research, CNR, S.S. Catania, via P. Gaifami 18, Catania, Italy; antonio.magri@ic.cnr.it, giovanni.tabbi@ic.cnr.ir, irina.naletova@ic.cnr.it, francesco.attanasio@ic.cnr.it

[2] Consorzio Interuniversitario per la Ricerca dei Metalli nei Sistemi Biologici, Via Ulpiani 27, Bari, Italy;

[3] Department of Chemical Sciences, University of Catania, Viale A. Doria 6, Catania, Italy; garena@unict.it

[#] AM and GT share the first authorship

* Correspondence: erizzarelli@unict.it; Tel.: +39 095 7385070 (E.R.); francesco.attanasio@ic.cnr.it (F.A.)

Table S1. Log β and pK values for the protonation of the $Ctr1_{(1-14)}$.

Species $[L_qH_r]$	log $\beta^{a,b}$	pK^b	log β^c	pK^c
qr				
11	9.64 (3)	9.64	9.84	9.84
12	17.27 (2)	7.63	17.30	7.46
13	24.18 (2)	6.91	24.23	6.93
14	30.47 (1)	6.29	30.46	6.23
15	36.21 (2)	5.74	36.24	5.78
16	40.39 (3)	4.17	40.27	4.03
17	43.21 (1)	2.82	43.20	2.92

[a]3σ in parentheses; [b]This work; [c]Reference 17
